# Effect of exercise intensity on redox biomarkers in healthy adults: A systematic review and meta-analysis of randomized clinical trials

**DOI:** 10.1371/journal.pone.0330185

**Published:** 2025-08-20

**Authors:** Flor Isela Torres-Rojo, Liliana Aracely Enríquez-del Castillo, Susana Aidée González-Chávez, Luis Alberto Flores-Olivares, Estefanía Quintana-Mendias, Claudia Esther Carrasco-Legleu

**Affiliations:** 1 Facultad de Ciencias Químicas, Universidad Autónoma de Chihuahua, Chihuahua, Chihuahua, México; 2 Facultad de Ciencias de la Cultura Física, Universidad Autónoma de Chihuahua, Chihuahua, Chihuahua, México; 3 Facultad de Medicina y Ciencias Biomédicas, Universidad Autónoma de Chihuahua, Chihuahua, México; Emory University School of Medicine, UNITED STATES OF AMERICA

## Abstract

**Purpose:**

This meta-analysis aimed to establish the effect of vigorous and non-vigorous exercise interventions on biomarkers of redox status in healthy adults.

**Materials and methods:**

Based on the PRISMA guidelines, we searched the following databases: Scopus, Web of Science, PubMed, Springer, Science Direct, Cochrane Library, Dialnet, Redalyc, and Lilacs for randomized controlled clinical trials investigating the effect of non-vigorous and vigorous chronic exercise in healthy adults, with the evaluation of antioxidants. The quality of evidence and risk of biases were assessed using the PEDro scale and version 2 of the Cochrane tool for risk of bias assessment in randomized trials (RoB2).

**Results:**

Seven randomized clinical trials evaluating nine training protocols were included (n=267). Individual evaluations demonstrated an increase in antioxidant capacity (I^2^=0%, Z=4.56, p<0.00001) and superoxide dismutase (I^2^=52%, Z=1.94, p=0.05), an antioxidant enzyme, and decrease in pro-oxidant (I^2^=0%, Z=5.91, p<0.0001); there was no significant difference in glutathione peroxidase (I^2^=81%, Z=0.50, p=0.62). The effect of vigorous interventions showed an increase in antioxidants (Z=2.44, I^2^=67%, p=0.01) and a decrease in oxidants (Z=5.44, I^2^=0%, p<0.00001), while in non-vigorous exercise, no significant differences were observed in redox status.

**Conclusions:**

The vigorous physical exercise presented better results on antioxidant and oxidative capacity compared to non-vigorous intensity training protocols in healthy people. Finding the optimal balance between exercise intensity and oxidative stress is crucial for maximizing the production of antioxidant enzymes, which can enhance physiological function and increase resistance to OS.

## 1. Introduction

Oxidative stress (OS) is the imbalance between oxidizing and antioxidant molecules with a greater inclination towards oxidants [1–4]. The elevation of reactive oxygen species (ROS), either due to overproduction or deficiencies in their elimination, modifies and affects different macromolecules such as lipids, proteins, and nucleic acids, generating cellular damage through necrotic and apoptotic processes [5]. The OS is recognized as a central mechanism in the aging process and in several pathologies [3,6].

Metabolic activity in biological systems involves the production of ROS, a cellular process necessary for regulating numerous signaling pathways and modulating vital processes such as energy production, cellular respiration, photosynthesis, and toxin metabolism, among others. Physiologically normal ROS levels are maintained by antioxidant mechanisms involving enzyme systems and antioxidant molecules, which establish a balance between oxidation and reduction reactions in the cell [7].

While it has been shown that exercise results in OS [8,9], regular training appears to have adaptations, which can reduce exercise-induced OS [10,11]. Physiological levels of reactive oxygen species are essential for optimal force generation in skeletal muscle. In contrast, high levels of ROS promote contractile dysfunction of skeletal muscle and promote muscle fatigue [12].

Antioxidant enzymes protect cells and tissues against OS during physical exercise, being the first level of antioxidant defense [6]. Catalase (CAT) plays a critical role in neutralizing hydrogen peroxide generated by increased metabolism and energy demand, and its function is especially relevant in tissues with high metabolic activity, such as skeletal muscle. Besides superoxide dismutase (SOD) contributes to the balance in the elimination of superoxide. At the same time, glutathione peroxidase (GPx) collaborates with glutathione reductase to recycle oxidized glutathione, keeping it active and participating in cellular detoxification processes [6,13–15]. Therefore, quantification of antioxidant enzymes could respond to an imbalance produced by the presence of free radicals, generating OS induced by the intensity of exercise [6].

Regular chronic exercise can stimulate the activation of antioxidant enzymes in muscle, blood cells, and plasma [3,4,9,15], and may even slow mitochondrial deterioration and delay apoptosis [16].

What is not yet fully understood is how the redox balance varies over time in response to a sustained increase in physical activity, particularly exercise-induced changes in markers of antioxidants and OS at different intensities.

It has been suggested that exercise-induced OS depends on its intensity [17,18]; however, heterogeneity among studies, including the diversity of populations studied, exercise protocols, and monitoring individual biomarkers of antioxidant enzymes, make it difficult to obtain conclusive data [17,18]. Additionally, the cellular mechanisms that explain the influence of exercise intensity on OS are still inconsistent and multidimensional [17].

The pathophysiological processes of adaptive phenomena and imbalances in the redox state generally refer to methodological differences in the different exercise protocols administered. The severity of an imbalance in the OS depends on several factors, including the intensity of exercise and the individual’s state of health [15,18].

The description of the mechanisms of redox homeostasis generated by the effect of exercise at different intensities in healthy individuals could establish the physiological basis to explain the mechanisms triggered by the impact of physical exercise in pathological conditions where the redox state is out of balance. Therefore, this systematic review with meta-analysis aims to establish the effect of vigorous and non-vigorous exercise interventions on biomarkers of redox status in healthy adults.

## Materials and methods

The systematic review was conducted according to the guidelines of the Preferred Reporting Items for Systematic Reviews and Meta-Analyses (PRISMA) [19].

### 2.1 Search strategy

We searched the following databases for articles: Scopus, Web of Science, PubMed, Springer, Science Direct, Cochrane Library, Dialnet, Redalyc, and Lilacs from January to February 2024. The search was carried out with the PICOS strategy using the following keywords in all possible combinations: “physical exercise”, “physical activity”, training, exercise, “oxidative stress index”, ROS, “reactive oxygen species”, “free radical”, catalase, SOD-1, “superoxide dismutase”, “glutathione peroxidase”, diet and supplementation, with the boolean operators AND, OR, and NOT; to broaden the sample, a citation backward search was also performed. The work was carried out independently by two researchers, FITR and LAEC.

### 2.2 Inclusion and exclusion criteria

The following inclusion criteria for selected articles were considered: (a) randomized controlled clinical trials investigating the effect of chronic physical exercise in healthy adult humans under 68 years of age; (b) studies published in English with no time restriction on publication; (c) that evaluated at least one of the antioxidants: total antioxidant capacity (TAC), CAT, GPx and/or SOD and any biomarker of OS. Exclusion criteria included: a) study subjects who had received supplementation and (b) studies that included sports practice or acute exercise sessions.

### 2.3 Selection criteria

**Fig 1:**
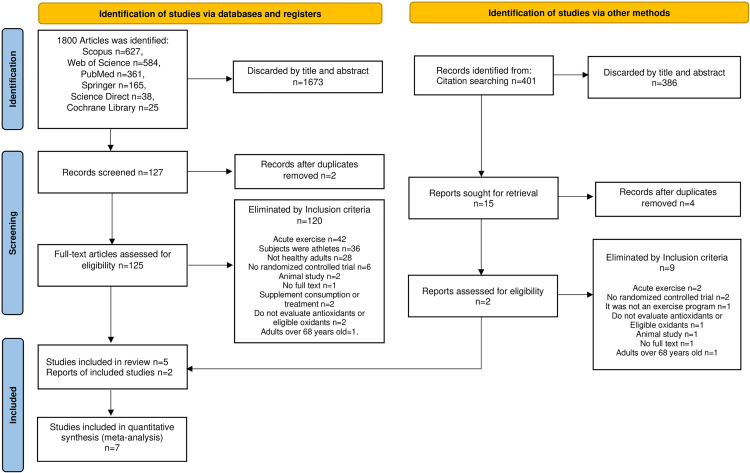
Flowchart showing the procedure used for study selection.

The selection process starting with reading the title and the abstract, eliminating the duplicates, then the text is reviewed. The reason for exclusion is listed in the Fig 1. Finally, the seven articles that meet the inclusion criteria. Table 1 shows the most essential characteristics and the results of the analysis of the selected articles.

**Table 1:** Characteristics of included studies in this meta-analysis.

Num	Reference and location	Participants	Exercise type and intensity	Exercise protocol	Specimen	Measurement technique	Results
**A1**	Kotowska et al., 2022/ Poland [23]	**TG:** n = 38; M; 21.1 ± 1.2 yr **CG:** n = 25; M; 21.3 ± 1.3 yr	Aerobic Vigorous	Swimming training program 12 wks/4 times per wk/90 min warm-up10- to 20-min	Plasma/ erythrocytes	Spectrophotometric	**Antioxidant: ↑**SOD **↓**GPx*
**A2**	Alikani et al., 2019/ Iran [24]	**TG:** Divided on: **1. YTG** n = 12; F; 22.1 ± 3 yr **CG:** n = 12; F; 23.2 ± 2 yr **2. ETG** **TG:** n = 12; F; 60.4 ± 3 yr **CG:** n = 12; F; 62.3 ± 3 yr	Anaerobic Vigorous	Progressive resistance training 12 wks/75% of 1RM/3 times per wk/120 min sets of 10 repetitions with six resistance movements in machine with 1–2 min rest period	Plasma	ELISA	↓ **YT oxidant:** MDA** ↑YT antioxidant: TAC* ↓ **ET oxidant:** MDA** ↑ **ET antioxidant:** TAC*
**A3**	Bunpo et al., 2016/ Thailand [25]	**TG:** n = 10; F&M; 24 ± 1.6 yr **CG:** n = 16; F&M; 22 ± 0.5 yr	Aerobic Non- vigorous	Outdoor running program 12 wks/at 65–75% HRmax/ 3 times per wk/30 min	Plasma/ erythrocytes	Spectrophotometric	↓ **Oxidant:** MDA **Antioxidant:** ↑TAS ↓SOD ↓ GPx ↓CAT
**A4**	Johnson et al., 2015/ Denmark [26]	**TG:** n = 11; 6M, 5F; 24.9 ± 1.0 yr **CG:** n = 12; 6M; 6F; 26.6 ± 0.7 yr	Aerobic Non- vigorous	Supervised bicycle training program 8 wks/at 65% HRmax/3–5 times per wk/60 min	Muscle biopsy	Spectrophotometric	**Antioxidant:** ↑SOD ↑CAT
**A5**	Soares et al., 2015/ Portugal [27]	**TG:** n = 31; M; 52.4 ± 9.1 yr **CG:** n = 26; M; 58.4 ± 10.2 yr	Aerobic and anaerobic Vigorous	Concurrent training program 16 wks/at 55–75% HRR/ 65–75% of 1RM/3 times per wk/ 60 min Two sets of 10–15 repetitions in the first 4 wks, and three sets of 10–15 repetitions in the following wks	Plasma	Spectrophotometric	**Oxidant:** ↓MDA* Antioxidant: ↑TAC*
**A6**	Azizbeigi et al., 2015/ Iran [28]	**TG:** Divided on: **1. MR:** n = 10; M; 20.8 ± 1.8 yr **2. HR:** n = 10; M; 20.5 ± 1.1 yr **CG:** n = 10; M 21.5 ± 2.2 yr	Anaerobic Non- vigorous Anaerobic Vigorous	Moderate intensity resistance training 8 wks/at 65–70% of 1RM/3 times per wk/120 min 3 sets of 10–12 repetitions with a 1–2 min rest period High intensity resistance training 8 wks/ at 85–90% of 1RM**/**3 times per wk/120 min 3- 6 repetitions with a 3–4 min rest period	Plasma/ erythrocytes	Spectrophotometric	**MR oxidant:** ↓MDA * MR antioxidant: ↑SOD ↑GPx * HR oxidant: ↓MDA* HR antioxidant: ↑SOD* ↓GPx *
**A7**	Azizbeigi et al., 2013/ Iran [29]	**TG:** n = 10; M; 21.2 ± 2.1 yr **CG:** n = 10; M; 23.3 ± 2.5 yr	Anaerobic Vigorous	Progressive Resistance Training 8 wks/50–80% of E1RM**/**3 times per wk/120 min 8–12 repetitions, three sets in each exercise, increased by approximately 5% each wk	Plasma/ erythrocytes	Spectrophotometric	**Oxidant: ↓**MDA* Antioxidant: ↑TAC ↑GPx ↑SOD*

A1-A7 = number of article selected; TG = Training Group; CG = Control Group; n = sample size; yr = age in years; M = Male; F = Female; wks = weeks; wk = week; NR = not reported; HRmax = maximum Heart Rate; HRR = Heart Rate Reserve; 1 RM = 1 maximum repetition; E1RM=1 Estimated Maximum Repetition; ETG = Elderly Training Group; YTG = Young Training Group; MR = Moderate Resistance; HR = High Resistance; MDA = Malondialdehyde; SOD = Superoxide Dismutase; GPx = Glutathione Peroxidase; CAT = Catalase; TAC = Total Antioxidant Capacity; TAS = Total Antioxidant Status; * = P ≤ 0.05; ** = P ≤ 0.001

### 2.4 Methodological quality and assessment of risk of bias

The methodological quality of the eligible studies was assessed using the PEDro scale [20]; the risk of bias of all included articles was carried out independently by two authors (FITR and LAEC) who assessed the quality of the current evidence for further comparison and discussion of possible discrepancies. We used version 2 of the Cochrane Risk of Bias Assessment Tool in Randomised Trials (RoB 2), which assesses five domains. Within each domain, raters answered one or more signaling questions. The responses led to judgments of “low risk of bias”, “some bias concerns”, or “high risk of bias” [21]. The results were schematized in Review Manager software (RevMan 5.4) [22].

### 2.5 Statistical analysis

Statistical analysis was performed by calculating the standardized mean difference (SMD) with a 95% confidence interval (CI) for antioxidants and biomarkers of OS reported in at least three studies. The value of I^2^ corresponds to the statistical heterogeneity of the included studies. If the I^2^ value is ≥ 50%, the random-effects model (REM) was used; otherwise, the fixed-effect model (FEM) was used [22,23]. This statistical analysis was performed in Rev Man version 5.4. and a significance of p ≤ 0.05 was considered.

### 2.6 Data extraction

Articles were arranged by number according to the year of publication, and an “A” was initially assigned to a consecutive sequence from A1 to A7 [24–30]. Physical exercise was classified as vigorous and non-vigorous based on the classification reported in each reviewed article, following the American College of Sports Medicine (ACSM) guidelines [31]. Vigorous exercise included intensities ranging from 60–89% (vigorous) to ≥90% (maximal), while non-vigorous exercise referred to moderate intensity protocols 40–59%. Only A1, A2, and A3 [24–26] provided numerical data on oxidants and antioxidants, while the other authors presented their results through bar graphs and so WebPlotDigitizer 4.7 was used to calculate the numerical value [32]. In A4 [27], the SOD and CAT values were squared to eliminate negative values and thus homogenize the results.

## Results

Of the 1,805 articles found, only five were selected following the selection procedure of the diagram presented in Fig 1. Two articles identified by backward search citation search that followed the same method for inclusion were added.

### 3.1 Assessment of methodological quality

**Fig 2:**
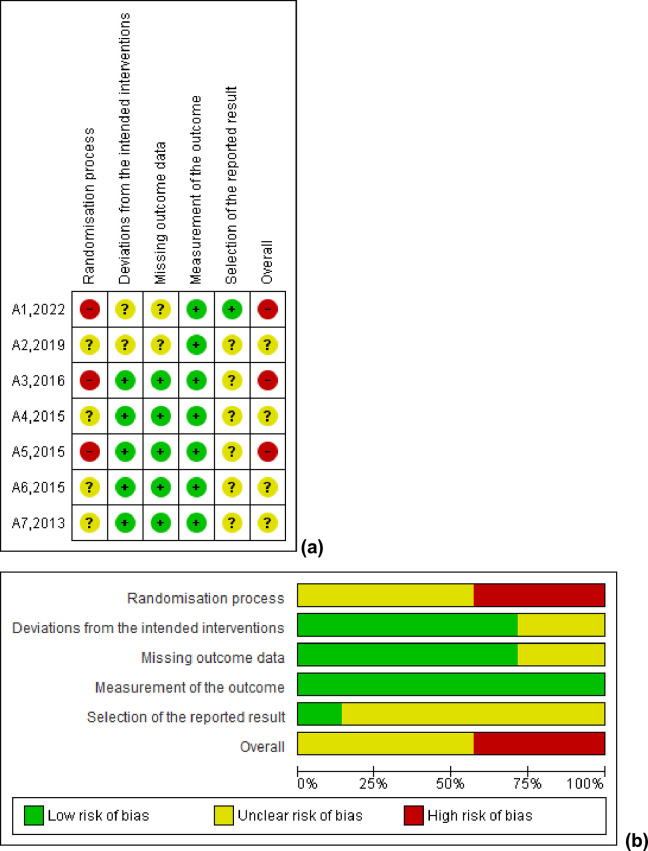
The risk of bias assessment. (a) Risk of bias summary: circle green low risk of bias; circle yellow unclear risk of bias; circle red high risk of bias; (b) risk of bias graph in percentage bias.

Table 2 shows the PEDro score of the included studies, classified as “moderate methodological quality”, with a score of 5. Item 1 of the PEDro scale is not included in the total score, as it is related to the external validity of the study. In contrast, items1, 5, 6, and 7 were not considered due to the limitations of studies that apply physical exercise protocols. The assessment of the risk of bias is shown in Fig 2. Where four of the included studies were classified with “some bias concerns” and three with “high risk of bias”. Only one study [25] specified the randomization technique performed, while three of the studies [24,25,28] reported participant losses. No studies reported the existence of a prospectively registered protocol.

**Table 2:** PEDro scores of included studies.

Criteria/study	A1 [24]	A2 [25]	A3 [26]	A4 [27]	A5 [28]	A6 [29]	A7 [30]
1. Random allocation	*	*	*	*	*	*	*
2. Blinding of allocation	1	1	1	1	1	1	1
3. Baseline similarity	0	0	0	0	0	0	0
4. Blinding of participants	1	1	1	1	1	1	1
5. Blinding of therapists	*	*	*	*	*	*	*
6. Blinding of evaluators	*	*	*	*	*	*	*
7. < 15% loss sample	*	*	*	*	*	*	*
8. Analysis by intention to treat	1	1	1	1	1	1	1
9. Difference between groups	1	1	1	1	1	1	1
10.Trend measurements central and variability	1	1	1	1	1	1	1
Total (0–10)	5	5	5	5	5	5	5

0 = the criterion is not satisfied; 1 = the criterion is satisfied; * = not evaluate.

### 3.2 Study characteristics

Four of the seven included articles were conducted on the Asian population [25,26,29,30] and the rest of the European population [24,27,28]. Most articles evaluated a single training protocol, while A2 and A6 [25,29] performed two protocols in the same study. Finally, nine training protocols from the seven included articles were reviewed.

Table 1 shows the main characteristics of the included protocols. In the case of A1 [24], the intensity of work applied was not specified, and was classified as vigorous because it consisted of the development of a swimming training program, which was reviewed in the Ainsworth Compendium of Physical Activities, where such activity corresponds to an intensity of 8.3 METs [33]. The results that were analyzed from the articles were only of the enzymatic activity of the antioxidants GPx, CAT, SOD, TAC, total antioxidant status (TAS), and the oxidant malonaldehyde (MDA). Five studies used spectrophotometric methods to measure oxidants and antioxidants in plasma [24,26,28–30]. study A2 [25], utilized ELISA for its measurements, while studyA4 [27] employed muscle biopsies analyzed through spectrophotometry and real-time PCR.

### 3.3 Effect of physical training

#### a) Antioxidant capacity.

**Fig 3:**
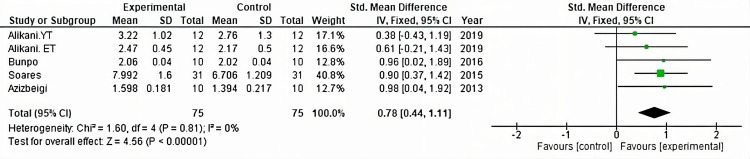
Forest plot showing the effect of exercise on antioxidant capacity. CI: confidence interval; IV: inverse variance; SD: standard deviation.

The antioxidant capacity was evaluated in A2, A5, and A7 [25,28,30] with the measurement of TAC, and A3 [26] determined TAS. A total of 75 subjects were analyzed. A significant increase in antioxidant capacity was found due to the effect of the applied vigorous or non-vigorous exercise programs with an I^2^ = 0% (Z = 4.56, p < 0.00001), as shown in Fig 3.

#### b) Oxidative capacity.

**Fig 4:**
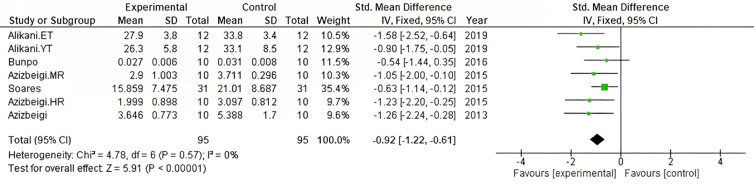
Forest plot showing the effect of exercise on oxidant capacity. CI: confidence interval; IV: inverse variance; SD: standard deviation.

Five of the reviewed studies, including 95 subjects in seven different training protocols [25,26,28–30], measured levels of MDA. The pooled data are shown in Fig 4. A high level of heterogeneity among data and a significant decrease in oxidant levels by the effect of physical training programs was observed (I^2^ = 0%, Z = 5.91, p < 0.00001).

#### c) Antioxidant enzymes.

**Fig 5:**
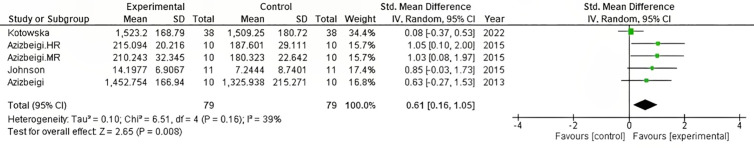
Forest plot showing the effect of exercise on SOD capacity. CI: confidence interval; IV: inverse variance; SD: standard deviation.

**Fig 6:**
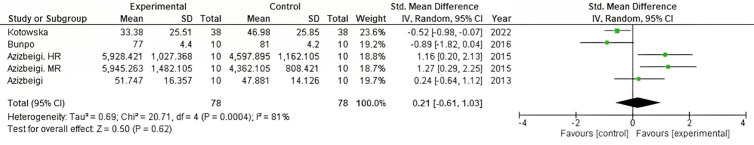
Forest plot showing the effect of exercise on GPx capacity. CI: confidence interval; IV: inverse variance; SD: standard deviation.

SOD evaluation was performed on 89 subjects, in six different exercise interventions [24,26,27,29,30], as shown in Fig 5. A heterogeneity of 39% was observed, and a significant increase in antioxidant enzymes was found (Z = 2.65, p = 0.008). The enzyme GPx was evaluated in four studies [24,26,29,30]; 78 subjects and five interventions were included. As expressed in Fig 6. no significant differences were observed in the effect of training (Z = 0.50, p = 0.62). CAT analysis with physical training could not be performed due to the limited number of articles evaluating this antioxidant (n = 21).

### 3.4 Effect of physical training by exercise intensity

#### a) Antioxidants.

The SOD, CAT, and GPx enzymes were analyzed individually with vigorous and non-vigorous intensity; however, the number of individuals in the articles available with the different intensities was not sufficient to be able to perform the analysis and obtain a conclusive result. In the case of SOD, evaluations with the protocols of non-vigorous (n = 31) and vigorous (n = 58) exercise was not significant, a similar result with GPx was found when evaluated with vigorous exercise (n = 58). Only two protocols measured GPx (n = 20) and CAT (n = 21) with non-vigorous exercise, and of the articles analyzed, no protocol was reported that evaluated CAT with vigorous exercise.

**Fig 7:**
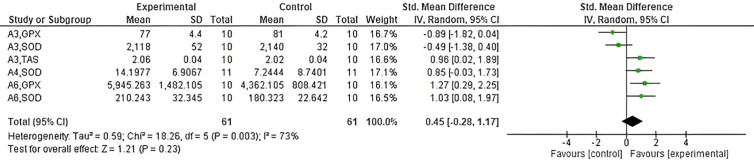
Forest plot showing the effect of no vigorous exercise on antioxidant capacity. CI: confidence interval; IV: inverse variance; SD: standard deviation.

**Fig 8:**
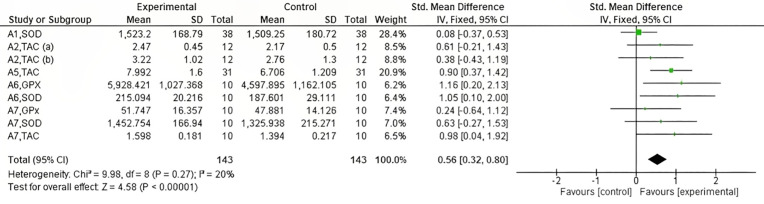
Forest plot showing the effect of vigorous exercise on antioxidant capacity. CI: confidence interval; IV: inverse variance; SD: standard deviation.

Of the studies analyzed with all antioxidants evaluated by exercise intensity, three training protocols [26,27,29], including a total sample size of 82 subjects, evaluated the effect of non-vigorous exercise on eight different antioxidants. As shown in Fig 7. no significant differences in antioxidants were found by non-vigorous intervention (Z = 1.21, p = 0.23). On the other hand, five training protocols [24,25,28–30], including an overall sample size of 143 subjects, evaluated the effect of vigorous exercise on nine different antioxidants. Contrary to those observed in non-vigorous, the vigorous training significantly modified the antioxidants (Z = 4.58, p = 0.00001, I^2^ = 20%), as shown in Fig 8.

#### b) Oxidants.

**Fig 9:**
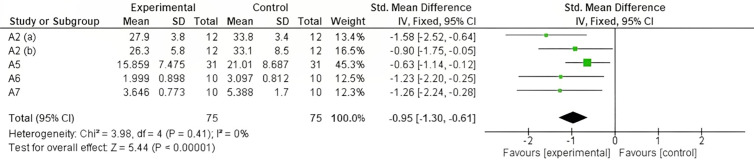
Forest plot showing the effect of vigorous exercise on oxidant levels capacity. CI: confidence interval; IV: inverse variance; SD: standard deviation.

Regarding the effect of vigorous exercise on oxidants, four applied protocols [25,28–30] were analyzed as shown in Fig 9. in which an increase in oxidants due to physical training was observed (Z = 5.44, p < 0.00001).

## Discussion

In this meta-analysis was evaluated the effect of exercise intensity on different redox biomarkers in healthy adults. This study excluded adults over 68 years because antioxidant enzyme activities are significantly reduced with age. [34,35]. Exercise interventions were classified as non-vigorous and vigorous according to the ACSM [31]. Following the analysis of results and a sensitivity analysis, certain studies were identified as outliers on the funnel plot and subsequently excluded to enhance the homogeneity of the data without affecting the overall-effect-size significance of the forest plot.

Our results show that physical activity increases total antioxidant capacity and decreases oxidative stress by reducing enzymatic MDA levels, thereby promoting the organism’s homeostasis through the reduction of ROS and, consequently, the OS [1,3]. This demonstrates exercise-induced responses characterized by increased antioxidants and decreased oxidants levels [36–38]. This analysis also indicates a differential response of antioxidant enzymes to vigorous and non-vigorous exercise, with SOD activity increasing in response with exercise. Moreover, vigorous exercise showed more favorable outcomes in both antioxidant capacity and oxidative stress markers compared to non-vigorous training protocols. In the individualized analysis of the antioxidants against exercise, without distinction of intensities, and concerning the evaluation of SOD, an increase is show towards the applied training programs (Z = 1.94 p = 0.05). When evaluating this enzyme by exercise intensity, only three articles assessed SOD under non-vigorous intensity [26,27,29] and three under vigorous intensity [24,29,30]. This represents a limited number of studies, making it difficult to draw reliable conclusions. It is important to note that SOD is one of the primary antioxidant enzymes involved in the defense against OS [6]. The lack of significant changes in SOD activity under vigorous exercise intensity may be attributed to its increased utilization in response to the elevated oxidative stress induced by the exercise.

In CAT, only 2 articles performed evaluations of this enzyme against an applied exercise program within the category of non-vigorous intensities [26,27], the sample size did not allow the analysis to be performed. In the case of vigorous intensities, no article performed evaluations of CAT; nevertheless, it is important to mention that this enzyme is essential in eliminating ROS, mainly hydrogen peroxide [7]. Different results have been presented in the literature. On the one hand, they are concordant with increased catalase due to exercise [39], while on the other, contradictory results observed no effects in this biomarker [40,41]. According to other authors [36], CAT appears unaltered by training effects after 13 weeks; they also reveal a higher CAT activity in trained subjects compared to untrained individuals wich may be explained by the fact that GPx and CAT have redundancy in their function to eliminate hydrogen peroxide as previous studies have determined that GPx is more efficient in low concentrations by depleting first, while CAT acts better in high concentrations [6,42]. No significant changes were detected in GPx levels. Upon comparison, it becomes evident that studies A1 [24] and A3 [26] negatively impact the effect size for GPx. Both studies followed 12 weeks protocols. This allows us to suggest that the protocols should be standardized by duration due to the probable use and depletion of the antioxidant enzymes or the metabolic effect on regulating the redox state [43].

In this meta-analysis, we were unable to assess GPx in response to non-vigorous exercise. However, GPx activity under vigorous exercise intensity was evaluated in three articles [24,29,30] with anon-significant result. This may suggest a depletion of this enzyme due to the high oxidative demand induced by intense exercise. It is also recognized that GPx functions more efficiently at low concentrations of reactive oxygen species, whereas CAT becomes more active under higher oxidative conditions [6,42]. It can even be a compensatory mechanism of the body because GPx is generally considered a protective molecule [42]. In general, oxidants and antioxidants were compared against the previously classified intensities. In the case of the comparisons of oxidants against non-vigorous training intensity, this was significant; however, we cannot have a clear conclusion due to the limited number of protocols that evaluated this intensity. The outcome observed in antioxidants with this same intensity (Fig 7.) is also without statistical significance; this may be because there is no energy utilization stimulus, which leads to an oxidative process that triggers an elevation in antioxidant enzymes as a compensatory mechanism in the studies were evaluated.

Similar results were obtained by other authors [37]. Where a non- intensive cycling program was applied, the systemic response of antioxidants and inflammatory biomarkers in healthy subjects was evaluated; they confirmed that moderate training in unaccustomed subjects does not increase oxidative damage. On the contrary, it represents a clear example of the adaptive stimulation of OS in the organism. Additionally, the authors state that the reduction in lipid peroxidation is parallel with the antioxidants evaluated, which can result in an efficient combination of oxidants and antioxidants. Furthermore, repeated exercise can induce a transient reduction of specific antioxidants, indicating a better redox balance [44].

It has even been suggested that OS is maintained during excessive training due to the hydrogen peroxide emission produced by mitochondria [43]. However, this increase in ROS during exercise is effectively neutralized by the antioxidant system, which includes SOD, CAT, and GPx, in healthy individuals [45]. Therefore, when evaluating vigorous exercise in relation to OS, significance was found in the elevation of antioxidants (Z = 2.44, p = 0.01) in the training programs analyzed (Fig 8). This may arise as a response to the elevation of ROS produced by the high energy demand generated at these intensities since the organism, by increasing respiration, introduces a greater amount of oxygen, with the consequent production of OS [6,46].

This interpretation is supported by text above is matched by the significance observed in the result obtained for oxidants with vigorous intensity (Z = 5.44, p < 0.00001) which occurs in healthy individuals due to the increase ROS (Fig 9). This may justify the response of the organism based on the homeostasis necessary to neutralize the oxidants produced. Other authors even suggest that increased DNA damage may be associated with increased exercise intensity in a dose-dependent manner, and that this damage may be transient and should not confer any adverse long-term health outcomes in the healthy individual or athlete [47].

A meta-analysis published in 2022 [48] on intracellular OS mentions the importance of the heterogeneity of different exercise protocols in clinical populations with unhealthy study participants. It also emphasizes that the indicators to identify OS may differ between populations and should be selected according to the specific effects of their diseases on oxidative metabolism. Therefore, it is essential to establish the exercise protocol and its specific characteristics for its favorable use concerning the homeostasis of the redox system.

It is important to note that a greater number of studies are required to evaluate the effect of OS at different intensities, so it is recommended that more research be carried out on the effect of these enzymes at different intensities in exercise programs, as the evidence is limited in healthy people.

## Conclusion

This meta-analysis suggests that vigorous exercise presented better results on antioxidant and oxidative capacity compared to non-vigorous intensity training protocols in healthy people. Finding the optimal balance between exercise intensity and oxidative stress is crucial for maximizing the production of antioxidant enzymes, which can enhance physiological function and increase resistance to OS. Exercise-induced hormesis in healthy people could describe the dose-response of metabolic challenge on redox status at different intensities and volumes of physical exercise and lead to better physiological function with increased resistance to OS to provide efficient cellular protective effect in other patients. Further research is needed to understand better the dose-response relationship of exercise-induced hormesis on redox status and physiological function.

## Supporting information

S1 File Checklist Prisma 2009 checklist.
(PDF)

S2 File Description of excluded records and reasons. (PDF)

S3 File Forest plot showing the effect of non-vigorous exercise on oxidant levels capacity. CI: confidence interval; IV: inverse variance; SD: standard deviation.
(PDF)

S4 File Forest plot showing the effect of exercise non-vigorous on SOD. CI: confidence interval; IV: inverse variance; SD: standard deviation.
(PDF)

S5 File Forest plot showing the effect of exercise vigorous on SOD. CI: confidence interval; IV: inverse variance; SD: standard deviation.
(PDF)

S6 File Forest plot showing the effect of exercise non-vigorous on GPx. CI: confidence interval; IV: inverse variance; SD: standard deviation.
(PDF)

S7 File Forest plot showing the effect of exercise vigorous on GPx. CI: confidence interval; IV: inverse variance; SD: standard deviation.
(PDF)

S8 File Forest plot showing the effect of exercise non-vigorous on CAT. CI: confidence interval; IV: inverse variance; SD: standard deviation.
(PDF)
