# Supplementary material for: Effect of exercise intensity on redox biomarkers in healthy adults: A systematic review and meta-analysis of randomized clinical trials
Source: PLoS One. 2025 Aug 20;20(8):e0330185. doi: 10.1371/journal.pone.0330185 (PMC12367122; doi:10.1371/journal.pone.0330185)
Supplement: S2 File — (PDF) [file pone.0330185.s002.pdf]

ID	Study	Included?	Reasons for exclusion
1	Effects of aquatic exercise on mental health, functional autonomy and oxidative stress in depressed elderly individuals: A randomized clinical trial.	No	Not healthy adults
2	Preliminary study: comparative effects of lung volume therapy between slow and fast deep-breathing techniques on pulmonary function, respiratory muscle strength, oxidative stress, cytokines, 6-minute walking distance, and quality of life in persons with COPD.	No	Not healthy adults
3	Infrared Low-Level Laser Therapy (Photobiomodulation Therapy) before Intense Progressive Running Test of High-Level Soccer Players: Effects on Functional, Muscle Damage, Inflammatory, and Oxidative Stress Markers-A Randomized Controlled Trial.	No	Subjects were athletes
4	Oxidative Stress Response's Kinetics after 60 Minutes at Different (30% or 100%) Normobaric Hyperoxia Exposures.	No	Acute exercise
5	Randomization to Treadmill Training Improves Physical and Metabolic Health in Association With Declines in Oxidative Stress in Stroke.	No	Adults over 68 years old
6	Effects of combined physical exercise training on DNA damage and repair capacity: role of oxidative stress changes.	Yes	Eligible
7	Walking Training Improves Systemic and Local Pathophysiological Processes in Intermittent Claudication.	No	Not healthy adults
8	Systemic oxidative stress is associated with lower aerobic capacity and impaired skeletal muscle energy metabolism in heart failure patients.	No	No randomized controlled trial
9	Acute exercise increases resistance to oxidative stress in young but not older adults.	No	Acute exercise
10	Exercise-Induced Reductive Stress Is a Protective Mechanism against Oxidative Stress in Peripheral Blood Mononuclear Cells.	No	Duplicate
11	Dynamic Resistance Training Improves Cardiac Autonomic Modulation and Oxidative Stress Parameters in Chronic Stroke Survivors: A Randomized Controlled Trial.	No	Not healthy adults

12	Uniform and prolonged changes in blood oxidative stress after muscle-damaging exercise.	No	Acute exercise
13	Salivary and Plasmatic Antioxidant Profile following Continuous, Resistance, and High-Intensity Interval Exercise: Preliminary Study.	No	Acute exercise
14	Supervised exercise training reduces oxidative stress and cardiometabolic risk in adults with type 2 diabetes: a randomized controlled trial.	No	Not healthy adults
15	Exercise as a protective mechanism against the negative effects of oxidative stress in first-episode psychosis: a biomarker-led study.	No	Not healthy adults
16	Effect of exercise on oxidative stress: a 12-month randomized, controlled trial.	No	Not healthy adults
17	Effect of exercise therapy on lipid profile and oxidative stress indicators in patients with type 2 diabetes.	No	Not healthy adults
18	High day-to-day and diurnal variability of oxidative stress and inflammation biomarkers in people with type 2 diabetes mellitus and healthy individuals.	No	Not healthy adults
19	Acute effects of intradialytic aerobic exercise on solute removal, blood gases and oxidative stress in patients with chronic kidney disease.	No	Not healthy adults
20	A Short-Term Resistance Training Circuit Improved Antioxidants in Sedentary Adults with Down Syndrome.	No	Not healthy adults
21	The Effect of Whole-Body Cryotherapy at Different Temperatures on Proinflammatory Cytokines, Oxidative Stress Parameters, and Disease Activity in Patients with Ankylosing Spondylitis.	No	Not healthy adults
22	The relationship of oxidative stress and cholesterol with dipping status before and after aerobic exercise training.	No	Not healthy adults
23	Carbon monoxide, skeletal muscle oxidative stress, and mitochondrial biogenesis in humans.	No	Acute exercise
24	Short-term cardiovascular physical programme ameliorates arterial stiffness and decreases oxidative stress in women with metabolic syndrome.	No	Not healthy adults

25	Effects of a simple prototype respiratory muscle trainer on respiratory muscle strength, quality of life and dyspnea, and oxidative stress in COPD patients: a preliminary study	No	Not healthy adults
26	Effect of C242T Polymorphism in the Gene Encoding the NAD(P)H Oxidase p22phox Subunit and Aerobic Fitness Levels on Redox State Biomarkers and DNA Damage Responses to Exhaustive Exercise: A Randomized Trial.	No	Acute exercise
27	Resistance training improves sleep quality, redox balance and inflammatory profile in maintenance hemodialysis patients: a randomized controlled trial.	No	Not healthy adults
28	Changes in vascular and inflammatory biomarkers after exercise rehabilitation in patients with symptomatic peripheral artery disease.	No	Not healthy adults
29	Oxidative stress is associated with decreased heart rate variability in patients with chronic kidney disease.	No	Not healthy adults
30	Exercise-intensity dependent alterations in plasma redox status do not reflect skeletal muscle redox-sensitive protein signaling.	No	Acute exercise
31	The effect of submaximal exercise preceded by single whole-body cryotherapy on the markers of oxidative stress and inflammation in blood of volleyball players.	No	Subjects were athletes
32	Effect of Regular Aerobic Activity in Young Healthy Athletes on Profile of Endothelial Function and Platelet Activity.	No	Subjects were athletes
33	Internet-delivered lifestyle physical activity intervention: limited inflammation and antioxidant capacity efficacy in overweight adults.	No	Not healthy adults
34	Xanthine oxidase activity is associated with risk factors for cardiovascular disease and inflammatory and oxidative status markers in metabolic syndrome: effects of a single exercise session.	No	Not healthy adults
35	In Healthy Young Men, a Short Exhaustive Exercise Alters the Oxidative Stress Only Slightly, Independent of the Actual Fitness.	No	Acute exercise
36	The Effect of a 12-Week Health Training Program on Selected Anthropometric and Biochemical Variables in Middle-Aged Women.	No	No randomized controlled trial

37	Changes in Antioxidant Defense Capability and Lipid Profile after 12-Week Low- Intensity Continuous Training in Both Cigarette and Hookah Smokers: A Follow-Up Study.	No	Not healthy adults
38	Differential Effect of Endurance Training on Mitochondrial Protein Damage, Degradation, and Acetylation in the Context of Aging.	Yes	Eligible
39	Disturbances in pro-oxidant-antioxidant balance after passive body overheating and after exercise in elevated ambient temperatures in athletes and untrained men.	No	Acute exercise
40	Prooxidant/Antioxidant Balance in Hypoxia: A Cross-Over Study on Normobaric vs. Hypobaric "Live High-Train Low".	No	Acute exercise
41	The effect of a 12-week moderate intensity interval training program on the antioxidant defense capability and lipid profile in men smoking cigarettes or hookah: a cohort study.	No	Not healthy adults
42	Insulin, catecholamines, glucose and antioxidant enzymes in oxidative damage during different loads in healthy humans.	No	Supplement consumption or treatment
43	Role of exercise intensities in oxidized low-density lipoprotein-mediated redox status of monocyte in men.	No	Acute exercise
44	MitoQ supplementation augments acute exercise-induced increases in muscle PGC1α mRNA and improves training-induced increases in peak power independent of mitochondrial content and function in untrained middle-aged men.	No	Acute exercise
45	Exercise training and detraining effects on body composition, muscle strength and lipid, inflammatory and oxidative markers in breast cancer survivors under tamoxifen treatment.	No	Supplement consumption or treatment
46	Acute hypoxia and exercise-induced blood oxidative stress	No	Acute exercise
47	Exercise-induced oxidative stress and hypoxic exercise recovery	No	Acute exercise
48	Antioxidant enzyme activities and malondialdehyde levels related to aging.	No	Do not evaluate antioxidants or eligible oxidants.

49	Normal adaptations to exercise despite protection against oxidative stress	No	Animal study
50	Changes in blood lipid peroxidation markers and antioxidants after a single sprint anaerobic exercise	No	Acute exercise
51	The Effect of Progressive Resistance Training on Oxidative Stress and Antioxidant Enzyme Activity in Erythrocytes in Untrained Men cronico	Yes	Eligible
52	Effects of Aerobic-, Anaerobic- and Combined-Based Exercises on Plasma Oxidative Stress Biomarkers in Healthy Untrained Young Adults	No	Acute exercise
53	Effect of Moderate and High Resistance Training Intensity on Indices of Inflammatory and Oxidative Stress	Yes	Eligible
54	Exercise coupled with dietary restriction reduces oxidative stress in male adolescents with obesity	No	Not healthy adults
55	The Effect of an Increased Training Volume on Oxidative Stress.	No	No randomized controlled trial
56	IL-6 and HSPA1A Gene Polymorphisms May Influence the Levels of the Inflammatory and Oxidative Stress Parameters and Their Response to a Chronic Swimming Training.	Yes	Eligible
57	Erythrocyte oxidative status after maximal aerobic test in wrestlers.	No	Acute exercise
58	Exercise-Induced Reductive Stress Is a Protective Mechanism against Oxidative Stress in Peripheral Blood Mononuclear Cells	No	Acute exercise
59	Acute effect of intense exercises on serum superoxide dismutase, catalase and malondialdehyde levels in soccer players	No	subjects were athletes
60	Time-course of changes in oxidative stress and antioxidant status responses following a soccer game.	No	subjects were athletes
61	Changes in the Blood Antioxidant Defense Capacity During a 24 Hour Run.	No	Acute exercise

62	Production of free radicals and catalase activity during acute exercise training in young men.	No	Acute exercise
63	The effect of regular long term training on antioxidant enzymatic activities.	No	subjects were athletes
64	Physical performance and antioxidant effects in triathletes.	No	subjects were athletes
65	Influence of exercise on oxidant stress products in elite Indian cyclists.	No	subjects were athletes
66	Oxidative stress in half and full ironman triathletes.	No	subjects were athletes
67	Antioxidant status of interval-trained athletes in various sports	No	Subjects were athletes
68	Exercise session promotes antioxidant changes in Brazilian soccer players.	No	Subjects were athletes
69	Relation between oxidative stress markers and antioxidant endogenous defences during exhaustive exercise.	No	Acute exercise
70	Habitual exercise induced resistance to oxidative stress.	No	Animal study
71	Pre-exercise antioxidant enzyme activities determine the antioxidant enzyme erythrocyte response to exercise.	No	No Full Text
72	Effect of aerobic and anaerobic metabolism on free radical generation swimmers.	No	subjects were athletes
73	Blood free radical antioxidant enzymes and lipid peroxides following long-distance and lactacidemic performances in highly trained aerobic and sprint athletes.	No	subjects were athletes
74	Oxidative Stress Response’s Kinetics after 60 Minutes at Different (30% or 100%) Normobaric Hyperoxia Exposures.	No	Acute exercise

75	The Influence of Circadian Rhythm on the Activity of Oxidative Stress Enzymes.	No	Acute exercise
76	The effect of acute intense exercise on activity of antioxidant enzymes in smokers and non-smokers.	No	Acute exercise
77	Effect of C242T polymorphism in the gene encoding the nad(P)h oxidase p22phox subunit and aerobic fitness levels on redox state biomarkers and dna damage responses to exhaustive exercise: A randomized trial	No	Acute exercise
78	Effect of different running exercise modalities on post-exercise oxidative stress markers in trained athletes.	No	subjects were athletes
79	Anaerobic exercise-induced activation of antioxidant enzymes in the blood of women and men.	No	Acute exercise
80	Exercise-intensity dependent alterations in plasma redox status do not reflect skeletal muscle redox-sensitive protein signaling	No	Acute exercise
81	Resistance-Trained Individuals Are Less Susceptible to Oxidative Damage after Eccentric Exercise.	No	Acute exercise
82	Exercise-Induced Reductive Stress Is a Protective Mechanism against Oxidative Stress in Peripheral Blood Mononuclear Cells.	No	Acute exercise
83	Heightened exercise-induced oxidative stress at simulated moderate level altitude vs. sea level in trained cyclists	No	subjects were athletes
84	The influence of different types of physical activity on the redox status of scuba divers.	No	Acute exercise
85	In Healthy Young Men, a Short Exhaustive Exercise Alters the Oxidative Stress Only Slightly, Independent of the Actual Fitness	No	Acute exercise
86	Application of a new oxidation-reduction potential assessment method in strenuous exercise-induced oxidative stress	No	Acute exercise

87	Assessment of eccentric exercise-induced oxidative stress using oxidation-reduction potential markers	No	Acute exercise
88	Postexercise impact of ice-cold water bath on the oxidant-antioxidant balance in healthy men	No	Acute exercise
89	Acute exercise increases resistance to oxidative stress in young but not older adults	No	Acute exercise
90	The effect of submaximal exercise preceded by single whole-body cryotherapy on the markers of oxidative stress and inflammation in blood of volleyball players	No	subjects were athletes
91	Changes in athlete's redox state induced by habitual and unaccustomed exercise	No	subjects were athletes
92	Acute effects of resistance exercise and intermittent intense aerobic exercise on blood cell count and oxidative stress in trained middle-aged women	No	Acute exercise
93	Cytokines and oxidative stress status following a handball game in elite male players	No	subjects were athletes
94	Increased oxidative stress blood markers in well-trained rowers following two thousand-meter rowing ergometer race	No	subjects were athletes
95	Generation of reactive oxygen species after exhaustive aerobic and isometric exercise	No	Acute exercise
96	Sex-Specific Relationships of Physical Activity and Sedentary Behaviour with Oxidative Stress and Inflammatory Markers in Young Adults	No	No randomized controlled trial
97	Effects of high-intensity interval training with hyperbaric oxygen	No	Acute exercise
98	Effect of a shock micro-cycle on biochemical markers in university soccer players	No	Subjects were athletes
99	The influence of continuous and interval aerobic training on the oxidative status of woman basketball players.	No	Subjects were athletes

100	Changes in systemic and subcutaneous adipose tissue inflammation and oxidative stress in response to exercise training in obese black African women	No	Not healthy adults
101	Ten marathons in ten days: Effects on biochemical parameters and redox balance.	No	Subjects were athletes
102	Precompetitional weight reduction modifies prooxidative-antioxidative status in judokas	No	Subjects were athletes
103	Biochemical status, oxidative and antioxidant responses after 3-month specific training in elite karate athletes	No	Subjects were athletes
104	Variations in oxidative stress markers in elite basketball players at the beginning and end of a season	No	Subjects were athletes
105	Body Composition, Lipid Profile, Adipokine Concentration, and Antioxidant Capacity Changes during Interventions to Treat Overweight with Exercise Programme and Whole-Body Cryostimulation	No	Not healthy adults
106	The effects of two fitness programs with different metabolic demands on oxidative stress in the blood of young females.	No	No randomized controlled trial
107	Age-related responses in circulating markers of redox status in healthy adolescents and adults during the course of a training macrocycle	No	Acute exercise
108	Training effects on ROS production determined by electron paramagnetic resonance in master swimmers	No	Subjects were athletes
109	Moderate exercise blunts oxidative stress induced by normobaric hypoxic confinement	No	Acute exercise
110	The effect of endurance exercise on both skeletal muscle and systemic oxidative stress in previously sedentary obese men	No	Not healthy adults
111	Whole-body cryostimulation and oxidative stress in rowers: The preliminary results	No	Subjects were athletes
112	Effect of rugby training on blood antioxidant defenses in able-bodied and spinal cord injured players	No	Subjects were athletes

113	Severe exercise and exercise training exert opposite effects on human neutrophil apoptosis via altering the redox status	No	Do not evaluate antioxidants or eligible oxidants
114	Altered oxidative stress in overtrained athletes	No	Subjects were athletes
115	Exercise-induced oxidative stress in overload training and tapering	No	Subjects were athletes
116	Lipid peroxidation, erythrocyte superoxide-dismutase activity and trace metals in young male footballers	No	Subjects were athletes
117	Relationship between physical activity and oxidative stress biomarkers in women	No	No randomized controlled trial
118	Effect of altitude training on the peroxidation and antioxidant enzymes in sportsmen	No	Subjects were athletes
119	Aerobic and anaerobic training effects on the antioxidant enzymes of the blood	No	Subjects were athletes
120	The Influence of Winter Swimming on Oxidative Stress Indicators in the Blood of Healthy Males	No	subjects were athletes
121	Systemic Response of Antioxidants, Heat Shock Proteins, and Inflammatory Biomarkers to Short-Lasting Exercise Training in Healthy Male Subjects	No	Acute exercise
122	Antioxidant status of interval-trained athletes in various sports.	No	Duplicate
123	Effect of altitude training on the peroxidation and antioxidant enzymes in sportsmen.	No	Subjects were athletes
124	Antioxidant status and oxidative stress at rest and in response to acute exercise in judokas and sedentary men.	No	subjects were athletes
125	Steady-state redox status in circulating extracellular vesicles: A proof-of-principle study on the role of fitness level and short-term aerobic training in healthy young males	No	Acute exercise

126	Six weeks of high intensity cycle training reduces H2O2 emission and increases antioxidant protein levels in obese adults with risk factors for type 2 diabetes.	No	Not healthy adults
127	Effects of an exercise-based lifestyle intervention on systemic markers of oxidative stress and advanced glycation endproducts in persons with type 2 diabetes: Secondary analysis of a randomised clinical trial.	No	Not healthy adults

Identification of studies via other methods: citation

1	Aging and exercise training in skeletal muscle: responses of glutathione and antioxidantenzyme systems	No	Animal study
2	Oxidative stress and anti-oxidant responses to regular resistance training in young and older adult women.	Yes	Eligible
3	The effect of regular exercise and massage on oxidant and antioxidant parameters	No	Acute exercise
4	Antioxidant enzyme activities and malondialdehyde levels related to aging.	No	It was not an exercise program
5	Effects of different exercise program on blood markers of oxidative stress in young women.	No	No Full Text
6	Tai chi improves oxidative stress response and DNA damage/repair in young sedentary females.	No	No randomized controlled trial
7	Effects of combined resistance and cardiovascular training on strength, power, muscle cross-sectional area, and endurance markers in middle-aged men.	No	Do not evaluate antioxidants or eligible oxidants.
8	Ascorbic acid supplementation does not alter oxidative stress markers in healthy volunteers engaged in a supervised exercise program.	Yes	Eligible
9	A prior high-intensity exercise bout attenuates the vascular dysfunction resulting from a prolonged sedentary bout J Phys Act Health. 2019;16:916–24.	No	Acute exercise

10	Effect of Tai Chi exercise on DNA damage, antioxidant enzymes, and oxidative stress in middle-age adults	No	No randomized controlled trial
11	Tai chi diminishes oxidative stress in Mexican older adults	No	Adults over 68 years old
12	The Effect of Progressive Resistance Training on Oxidative Stress and Antioxidant Enzyme Activity in Erythrocytes in Untrained Men	No	Duplicate
13	Effect of Moderate and High Resistance Training Intensity on Indices of Inflammatory and Oxidative Stress	No	Duplicate
14	IL-6 and HSPA1A Gene Polymorphisms May Influence the Levels of the Inflammatory and Oxidative Stress Parameters and Their Response to a Chronic Swimming Training. Cronico	No	Duplicate
15	Effects of combined physical exercise training on DNA damage and repair capacity: role of oxidative stress changes.	No	Duplicate
