# Supplementary material for: Effect of exercise intensity on redox biomarkers in healthy adults: A systematic review and meta-analysis of randomized clinical trials
Source: PLoS One. 2025 Aug 20;20(8):e0330185. doi: 10.1371/journal.pone.0330185 (PMC12367122; doi:10.1371/journal.pone.0330185)
Supplement: S3 File — CI: confidence interval; IV: inverse variance; SD: standard deviation. (PDF) [file pone.0330185.s003.pdf]

	Experimental			Control				Std. Mean Difference	Std. Mean Difference
**Study or Subgroup**	**Mean**	**SD**	**Total**	**Mean**	**SD**	**Total**	**Weight**	**IV, Fixed, 95% CI**	**IV, Fixed, 95% CI**
A3	0.027	0.006	10	0.031	0.008	10	52.8%	-0.54 [-1.44, 0.35]	
A6	2.9	1.003	10	3.711	0.296	10	47.2%	-1.05 [-2.00, -0.10]	
**Total (95% CI)**			**20**			**20**	**100.0%**	**-0.78 [-1.43, -0.13]**	

Heterogeneity: Chi² = 0.58, df = 1 (P = 0.45); I² = 0%
Test for overall effect: Z = 2.35 (P = 0.02)

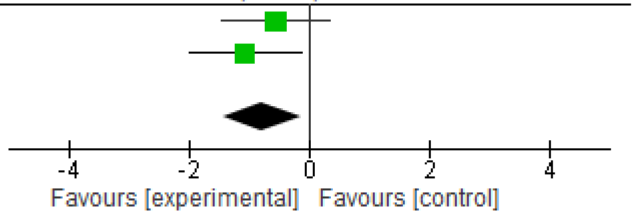

S3 File. Forest plot showing the effect of non-vigorous exercise on oxidant capacity.

CI: confidence interval; IV: inverse variance; SD: standard deviation.
