# Supplementary material for: Effect of exercise intensity on redox biomarkers in healthy adults: A systematic review and meta-analysis of randomized clinical trials
Source: PLoS One. 2025 Aug 20;20(8):e0330185. doi: 10.1371/journal.pone.0330185 (PMC12367122; doi:10.1371/journal.pone.0330185)
Supplement: S4 File — CI: confidence interval; IV: inverse variance; SD: standard deviation. (PDF) [file pone.0330185.s004.pdf]

	Experimental			Control				Std. Mean Difference		Std. Mean Difference
Study or Subgroup	Mean	SD	Total	Mean	SD	Total	Weight	IV, Random, 95% CI	Year	IV, Random, 95% CI
Azizbeigi.MR	210.243	32.345	10	180.323	22.642	10	31.9%	1.03 [0.08, 1.97]	2015	
Johnson	3.768	2.628	11	2.692	2.956	11	34.7%	0.37 [-0.47, 1.21]	2015	
Bunpo	2,118	52	10	2,140	32	10	33.4%	-0.49 [-1.38, 0.40]	2016	
**Total (95% CI)**			**31**			**31**	**100.0%**	**0.29 [-0.55, 1.13]**		

Heterogeneity: $Tau^2 = 0.34$; $Chi^2 = 5.28$, df = 2 (P = 0.07); $I^2 = 62\%$
Test for overall effect: Z = 0.69 (P = 0.49)

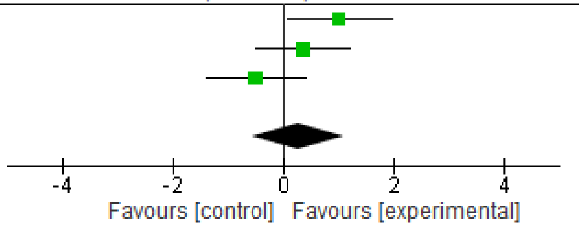

S4 File. Forest plot showing the effect of exercise non vigorous on SOD

CI: confidence interval; IV: inverse variance; SD: standard deviation
