# Supplementary material for: Effect of exercise intensity on redox biomarkers in healthy adults: A systematic review and meta-analysis of randomized clinical trials
Source: PLoS One. 2025 Aug 20;20(8):e0330185. doi: 10.1371/journal.pone.0330185 (PMC12367122; doi:10.1371/journal.pone.0330185)
Supplement: S5 File — CI: confidence interval; IV: inverse variance; SD: standard deviation. (PDF) [file pone.0330185.s005.pdf]

	EXPERIMENTAL			CONTROL				Std. Mean Difference	
Study or Subgroup	Mean	SD	Total	Mean	SD	Total	Weight	IV, Fixed, 95% CI	Year
Azizbeigi	1,325.938	215.271	10	1,452.754	166.94	10	16.8%	-0.63 [-1.53, 0.27]	2013
Azizbeigi.HR	187.601	29.111	10	215.094	20.216	10	15.3%	-1.05 [-2.00, -0.10]	2015
Kotowska	1,509.25	180.72	38	1,523.2	168.79	38	67.9%	-0.08 [-0.53, 0.37]	2022
**Total (95% CI)**			**58**			**58**	**100.0%**	**-0.32 [-0.69, 0.05]**	

Heterogeneity: Chi² = 3.83, df = 2 (P = 0.15); I² = 48%

Test for overall effect: Z = 1.69 (P = 0.09)

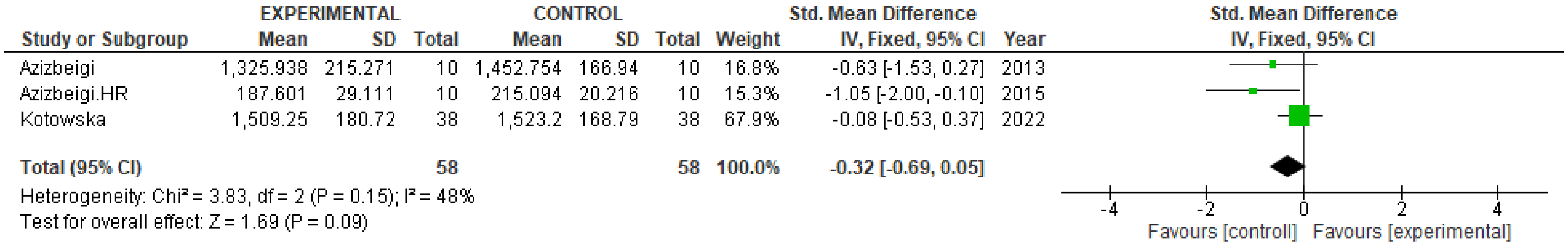

S5 File. Forest plot showing the effect of exercise vigorous on SOD

CI: confidence interval; IV: inverse variance; SD: standard deviation
