# Supplementary material for: Effect of exercise intensity on redox biomarkers in healthy adults: A systematic review and meta-analysis of randomized clinical trials
Source: PLoS One. 2025 Aug 20;20(8):e0330185. doi: 10.1371/journal.pone.0330185 (PMC12367122; doi:10.1371/journal.pone.0330185)
Supplement: S6 File — CI: confidence interval; IV: inverse variance; SD: standard deviation. (PDF) [file pone.0330185.s006.pdf]

	Experimental			Control				Std. Mean Difference	
**Study or Subgroup**	**Mean**	**SD**	**Total**	**Mean**	**SD**	**Total**	**Weight**	**IV, Random, 95% CI**	**Year**
Azizbeigi.MR	5,945.263	1,482.105	10	4,362.105	808.421	10	49.7%	1.27 [0.29, 2.25]	2015
Bunpo	77	4.4	10	81	4.2	10	50.3%	-0.89 [-1.82, 0.04]	2016
**Total (95% CI)**			**20**			**20**	**100.0%**	**0.18 [-1.93, 2.30]**	

Heterogeneity: Tau² = 2.10; Chi² = 9.83, df = 1 (P = 0.002); I² = 90%
Test for overall effect: Z = 0.17 (P = 0.86)

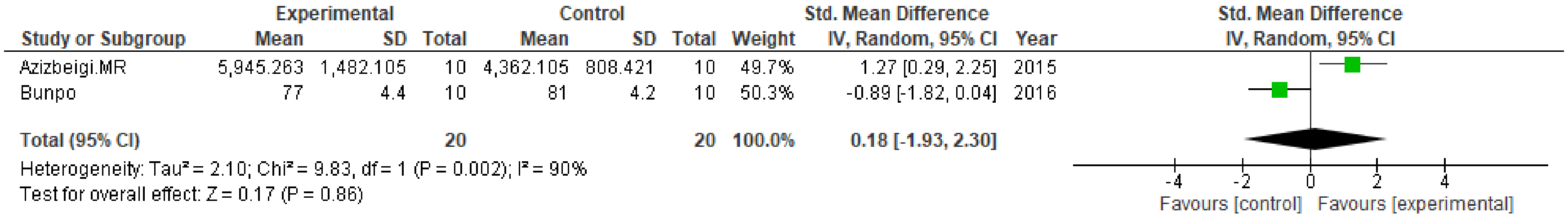

S6 File. Forest plot showing the effect of exercise non vigorous on GPx

CI: confidence interval; IV: inverse variance; SD: standard deviation
