# Supplementary material for: Effect of exercise intensity on redox biomarkers in healthy adults: A systematic review and meta-analysis of randomized clinical trials
Source: PLoS One. 2025 Aug 20;20(8):e0330185. doi: 10.1371/journal.pone.0330185 (PMC12367122; doi:10.1371/journal.pone.0330185)
Supplement: S7 File — CI: confidence interval; IV: inverse variance; SD: standard deviation. (PDF) [file pone.0330185.s007.pdf]

Study or Subgroup	Experimental Mean	Experimental SD	Experimental Total	Control Mean	Control SD	Control Total	Weight	Std. Mean Difference IV, Random, 95% CI	Year
Azizbeigi	51.747	16.357	10	47.881	14.126	10	31.5%	0.24 [-0.64, 1.12]	2013
Azizbeigi.HR	5,928.421	1,027.368	10	4,597.895	1,162.105	10	30.0%	1.16 [0.20, 2.13]	2015
Kotowska	33.38	25.51	38	46.98	25.85	38	38.4%	-0.52 [-0.98, -0.07]	2022
**Total (95% CI)**			**58**			**58**	**100.0%**	**0.22 [-0.77, 1.22]**	

Heterogeneity: $Tau^2 = 0.62$; $Chi^2 = 10.43$, $df = 2$ ($P = 0.005$); $I^2 = 81\%$
Test for overall effect: $Z = 0.44$ ($P = 0.66$)

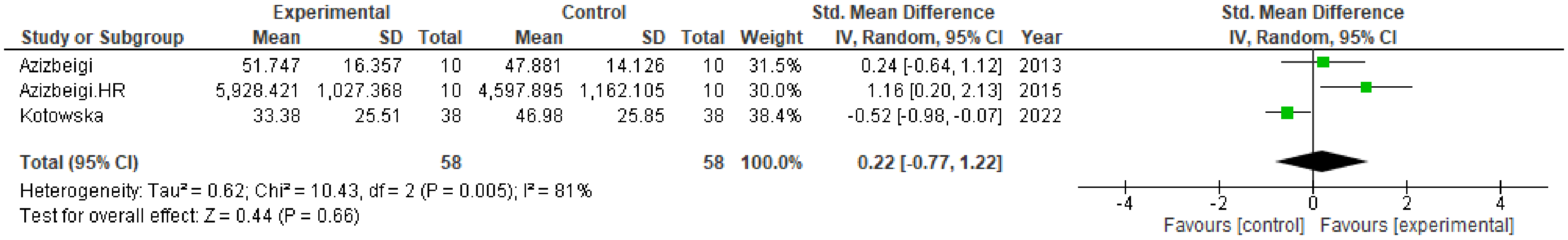

S7 File. Forest plot showing the effect of exercise vigorous on GPx

CI: confidence interval; IV: inverse variance; SD: standard deviation
