# Supplementary material for: Effect of exercise intensity on redox biomarkers in healthy adults: A systematic review and meta-analysis of randomized clinical trials
Source: PLoS One. 2025 Aug 20;20(8):e0330185. doi: 10.1371/journal.pone.0330185 (PMC12367122; doi:10.1371/journal.pone.0330185)
Supplement: S8 File — CI: confidence interval; IV: inverse variance; SD: standard deviation. (PDF) [file pone.0330185.s008.pdf]

	Experimental			Control				Std. Mean Difference	
**Study or Subgroup**	**Mean**	**SD**	**Total**	**Mean**	**SD**	**Total**	**Weight**	**IV, Random, 95% CI**	**Year**
Johnson	4,476.9317	395.7611	11	505.391	76.6193	11	49.0%	13.40 [8.95, 17.85]	2015
Bunpo	75	1.5	10	79	2.6	10	51.0%	-1.80 [-2.88, -0.73]	2016
**Total (95% CI)**			**21**			**21**	**100.0%**	**5.64 [-9.26, 20.54]**	

Heterogeneity: Tau² = 112.93; Chi² = 42.38, df = 1 (P < 0.00001); I² = 98%
Test for overall effect: Z = 0.74 (P = 0.46)

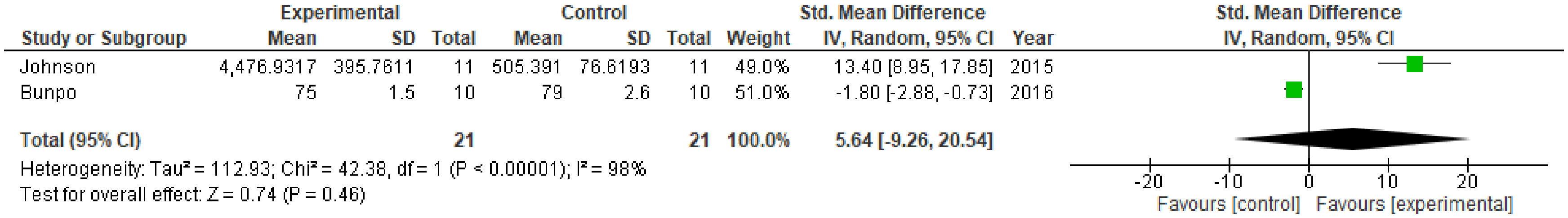

S8 File. Forest plot showing the effect of exercise non vigorous on CAT

CI: confidence interval; IV: inverse variance; SD: standard deviation
